# Hearing Aids: What Works Well and What Can Be Improved

**DOI:** 10.1007/s10162-026-01031-5

**Published:** 2026-02-13

**Authors:** Brian C. J. Moore

**Affiliations:** Cambridge Hearing Group, Department of Psychology, University of Cambridge, Downing Street, Cambridge, CB2 3EB UK

**Keywords:** Hearing aids, Occlusion effect, Closedness of fitting, Amplitude compression, Feedback cancellation, Directional microphones, Noise reduction

## Abstract

This paper evaluates the current performance of hearing aids, based on research findings and my experiences with hearing aids. The type of acoustic coupling to the ear is important. The fitting can be “closed” (sealing the ear canal), but this can lead to the occlusion effect; the user’s own voice sounds too loud or too boomy. Alternatively, the fitting can be open (the eartip has a vent). This alleviates the occlusion effect, but it introduces comb-filtering (heard as perceptual coloration) and leads to little or no gain at low frequencies. Also, the highest frequency at which useful gain can be achieved is often about 5 kHz, which is lower than optimal. While acoustic feedback cancellation systems have improved markedly, they can still introduce artifacts and impair sound quality, especially for music. Hearing aids use multi-channel amplitude compression to compensate for the reduced dynamic range of hearing-impaired people, but they often fail to restore the audibility of soft sounds, especially at high frequencies, and the amount of compression is often limited (and less than indicated by the manufacturers’ fitting software), leading to loudness discomfort (and sometimes reduced speech intelligibility) at high sound levels. Also, compression systems introduce cross-modulation, impairing sound quality. Most hearing aids incorporate directional processing and noise-reduction systems intended to improve the ability to understand speech in noisy situations. These systems can be effective with a closed fitting, but much of the benefit is lost with an open fitting because of leakage of background sounds through the vent.

## Introduction

 Hearing aid technology has changed markedly since the introduction of digital hearing aids in the 1980s. Many new forms of signal processing for hearing aids have been introduced. However, hearing aids are still far from returning hearing to “normal.” This limitation stems partly from the fact that some of the consequences of sensorineural hearing loss, such as reduced frequency selectivity [1], are not directly compensated by hearing aids; the effects of reduced frequency selectivity cannot be “undone” by processing in a hearing aid. To understand this, consider the excitation pattern produced by a 1-kHz sinusoidal tone; this pattern will be broader in a hearing-impaired cochlea than in a normal cochlea, and there is no way of processing the signal to make the excitation pattern sharper in the impaired ear. Another example of an effect that cannot be “undone” is cochlear synaptopathy, the loss of synapses between inner hair cells and primary auditory neurons that can occur following noise exposure [2–4] or with increasing age [5, 6]. The perceptual consequences of cochlear synaptopathy are uncertain, but it may lead to more “noisy” coding of auditory signals and hence to a reduced ability to understand speech in noisy situations [7, 8].

Despite these inherent limitations in what hearing aids can achieve, they can, in principle, compensate for some of the effects of sensorineural hearing loss, for example, reduced audibility and reduced dynamic range [9]. They can also, to some extent, improve the ability to understand speech in noisy situations. However, in my opinion, there is room for improvement in several aspects of hearing aid performance. For each aspect considered, I give a score out of 10 indicating my opinion about current performance. A score of 1 would indicate that a specific aspect is very poor indeed and is in considerable need of improvement, while a score of 10 would indicate that a specific aspect is the best that is likely to be achievable. The score is based on a composite of perceptual data from the literature, technical measurements (both published and my own), and my own personal experiences. Suggestions for improving performance are also given.

## Styles of Hearing Aids and Open Versus Closed Fittings

**Fig. 1:**
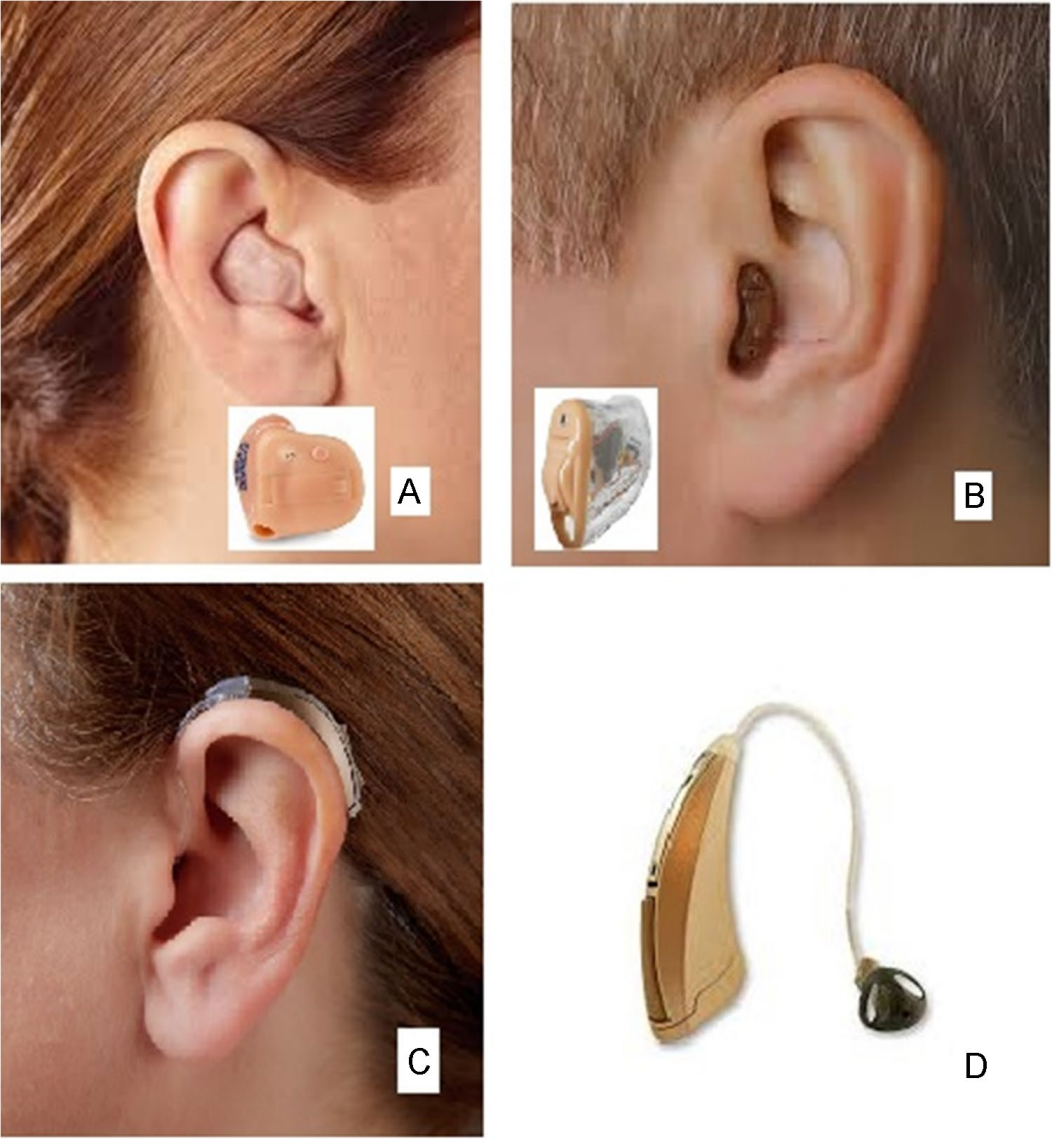
Illustration of the most common styles of hearing aid. From [10], by permission of the authors. The insets in panels **A** and **B** show the appearance of the aids when not inserted in the ear canal.

Figure 1 shows the most common hearing aid styles. When all of the components are housed in a single case or shell, this is called “in the ear” (ITE) if the shell is partly in the bowl of the pinna (Fig. 1A) or “completely in the canal” (CIC) if the shell fits completely in the ear canal (Fig. 1B). The most common style is “behind the ear” (BTE) (Fig. 1C), where the shell is placed behind the pinna and the microphones are just above the pinna. The miniature loudspeaker that generates the sound is called, counterintuitively, the receiver. In the “receiver-in-the-ear” (RITE) type of BTE, the receiver is inside the ear canal (Fig. 1D), but in some BTE aids, the receiver is in the BTE part and the sound is fed to the ear canal via a tube. The BTE style does not necessarily require a custom-made earmold or shell, whereas the ITE and CIC styles usually require a custom-made shell.

An important factor affecting the performance of hearing aids is the type of acoustic coupling to the ear. The fitting can be “closed” (sealing the ear canal). However, this can lead to the occlusion effect whereby the user’s own voice sounds too loud or too boomy and self-generated sounds like chewing are too loud. The effect occurs because low-frequency self-generated sounds from speaking or chewing are radiated into the ear canal via vibrations of the flexible outer portion of the ear canal [11]. When the ear canal is open, these sounds largely leak out of the ear canal. However, when the ear canal is closed by a hearing aid, the sound is trapped in the ear canal and creates the occlusion effect. This effect is often bothersome for people with mild hearing loss at low frequencies, which is common.

An alternative is to use an open fitting, where the shell of the hearing aid or the eartip (the part of a BTE hearing aid that goes into the ear canal) has a vent or opening, as in Fig. 1D. This alleviates the occlusion effect because it allows the low-frequency sound generated by vibration of the ear canal walls to escape. However, it has several undesirable effects:

- Interaction of the sound passing through the vent and the (delayed) sound delivered by the hearing aid leads to undesirable ripples in the frequency response (comb-filtering) [12, 13]. This leads to a perception that the sound is “colored” and it degrades sound quality [14, 15]. Comb-filtering is most prominent over the frequency range where the level in the ear canal of the (direct) sound passing through the vent is similar to the level of the delayed sound produced by the hearing aid. For a typical hearing-impaired person with a sloping hearing loss (greater at high frequencies), comb-filtering is most prominent for frequencies from about 500 to 1000 Hz, because the output level provided by the hearing aid is similar to the level leaking through the vent at those frequencies. However, the gain provided by most hearing aids decreases with increasing input level (as discussed later in this paper), so at high input levels, comb-filtering may occur over a wide frequency range [16]. Note that the effects of comb-filtering cannot be compensated by fixed linear filtering, because the pattern of comb-filtering varies with the input level.
- It is difficult to produce gain at low frequencies because low-frequency sounds generated by the hearing aid leak out of the ear canal. In practice, the gain falls off for frequencies below 1 kHz, and it is difficult to produce any positive gain for frequencies below 0.5 kHz. For people with near-normal low-frequency hearing, low frequencies may be heard via sound leaking through the vent, in which case the lack of amplification provided by the hearing aid is unimportant. However, for those with mild hearing loss at low frequencies, the lack of amplification may lead to a “tinny” or “thin” sound quality. Those with moderate or severe hearing loss at low frequencies would usually receive a closed fitting, so the issue does not arise for them. For sounds that are streamed wirelessly to the hearing aid (as discussed later in this paper), the lack of low-frequency amplification can lead to a “tinny” or “thin” sound quality regardless of the degree of hearing loss at low frequencies.
- The benefits obtained from directional processing and other forms of noise reduction (which are discussed later in this paper) are reduced, but not entirely lost, because background sounds from all directions leak through the vent [17].

There are “degrees” of openness [18]. A very small vent in the earmould or eartip may reduce the occlusion effect to some extent while also reducing the undesirable effects of a fully open fitting. However, this represents a compromise. Most people with mild-to-moderate sloping hearing loss (greater loss at high than at low frequencies) get fittings that are fully or partially open [19]. This largely avoids the occlusion effect but limits the benefit of directional microphones or noise reduction. The use of a more closed fitting can increase the benefit of directional microphones or noise reduction at the cost of an increase in the occlusion effect. It seems appropriate to use the smallest vent that leads to an acceptable occlusion effect.

## Effectiveness of Hearing Aids in Amplifying Sounds

A basic requirement for hearing aids is to improve audibility via amplification over a wide frequency range. However, the highest frequency at which useful gain can be achieved is often about 5 kHz [20]. Although some modern hearing aids can apply amplification for frequencies up to 8 or even 10 kHz [21, 22], the gains that can be achieved for frequencies above 5 kHz are often below the target gains prescribed by validated fitting methods for listeners with more than mild hearing losses [23–25]. The gains may be limited by the receiver characteristics, the available driving voltage, the manufacturer’s fitting software, or, most commonly, by acoustic feedback. Although most hearing aids incorporate feedback cancellation systems (as described below), the added stable gain that they provide is limited [26].

Several studies using a device that employs a transducer mounted on the eardrum and can provide high gain for frequencies up to 10 kHz (the Earlens [27]) have suggested that a more extended high-frequency response is beneficial both for speech intelligibility, reducing the reception threshold for speech in spatially separated competing speech by 1.3 dB on average [28], and sound quality, although the improvements in sound quality were modest [29–31]. However, studies using simulated hearing aids have not always revealed benefits of an extended high-frequency response [32]. It is likely that such benefits are obtained only when the gain at high frequencies is set appropriately [33, 34].

As noted above, an open fitting makes it difficult to produce gain at low frequencies (the Earlens device is an exception to this [35]). Many modern hearing aids allow “streaming” of sounds from devices such as mobile (cell) phones (as described later in this paper), and for streamed sounds, the lack of low frequencies can lead to poor sound quality [15]. In principle, the receivers used in hearing aids can produce an extended low-frequency response when a closed fitting is used [36], although they may not have a sufficient output for people with severe low-frequency hearing loss.

It may well be the case that the benefit of an extended high-frequency response depends on also having an extended low-frequency response, and vice versa [15, 31]; in other words, the tonal balance needs to be appropriate. An extended high-frequency response may lead to a tinny sound quality when low frequencies are not adequately amplified.

Overall, I rate the effectiveness of conventional hearing aids in providing amplification over a wide frequency range as 6/10, although the effectiveness will depend on the degree of hearing loss of the user; it may be possible to make high frequencies audible for people with mild hearing loss at high frequencies, but not for people with more severe loss. A higher rating, perhaps 9/10, is appropriate for “direct drive” systems such as the Earlens device and theoretically for middle-ear implants [37]. However, such systems are expensive because they require the involvement of an otolaryngologist and they require custom-made parts. Also, direct-drive systems require regular cleaning of the ear canal and eardrum by a specialist, which increases the “bother” of ownership.

## Amplitude Compression (Automatic Gain Control, AGC)

Almost all hearing aids incorporate some form of frequency-dependent amplitude compression to squeeze the wide range of sound levels encountered in everyday life into the limited dynamic range of the hearing-impaired ear [38, 39]. This limited dynamic range is also described in terms of “loudness recruitment”; if the level of a sound is progressively increased from a below-threshold value, once the sound level exceeds the detection threshold, the loudness grows more rapidly than normal, and when the input level reaches 90 to 100 dB SPL, the loudness approaches its “normal” value [40, 41]. Hence, the dynamic range between the threshold level and the highest comfortable level is smaller than normal. Since the dynamic range tends to decrease as the hearing loss increases, and hearing loss varies with frequency, the sound is often filtered into “channels” or frequency bands, and amplitude compression is applied independently in each channel. This is sometimes called multi-channel automatic gain control (AGC) or just multi-channel compression.

The fitting software of most manufacturers allows gains at each of several center frequencies to be set differently for low input levels (typically 50 dB SPL) and high input levels (typically 80 dB SPL). The difference in gain for the two levels determines the compression ratio (CR) at a given frequency. The CR is defined as the ratio (change in input level/change in output level). For example, if the input level is increased from 50 to 80 dB SPL, an increase of 30 dB, and the output level increases by 15 dB, the CR is 2. Hearing aid users with small dynamic ranges may require CRs of 10 or even more [25]. However, many hearing aid manufacturers limit the CR to 3 or less. This is partly based on evidence that CRs above 3 can have detrimental effects on speech intelligibility and sound quality [42, 43]. However, such deleterious effects have been shown mainly for compression systems in which the gain changes rapidly in response to changes in the level of the input signal (so-called fast compression, which is discussed below). To my knowledge, there is no compelling evidence that CRs above 3 should not be used in combination with slow-acting compression.

Another factor is that, in my experience, the obtained CRs are less than the CRs programmed using the manufacturer’s software. This applies both when the CRs are measured using artificial test signals, such as sinewaves or bands of noise, and when the CRs are measured using running speech presented over a range of levels. The lower-than-required CRs do not allow weak sounds to be made audible while preventing intense sounds from being uncomfortably loud [44]. Although output limiting may partially alleviate loudness discomfort for high input levels, some hearing aid users still complain about loudness discomfort, especially at live music events [44]. Also, the fitting software of some manufacturers does not allow gains to be set to negative values, even for high input levels. The intelligibility of speech in background sounds can decrease when the overall level exceeds a certain value, which is called the “rollover” effect [45]. Positive hearing aid gains for speech at high levels result in reduced intelligibility for some users [46].

Another aspect of AGC systems is compression speed. Hearing aids vary in how rapidly the gain changes in response to changes in input level. The time taken to reduce the gain in response to a sudden increase in sound level is characterized by the attack time, while the time taken to increase the gain in response to a sudden decrease in level is characterized by the release time [39]. The attack and release times vary across manufacturers, and some allow compression speed to be programmed. Many manufacturers use systems with multiple time constants (usually proprietary and “secret”). In theory, loudness recruitment is primarily caused by the loss of fast-acting compression in the cochlea [47], so fast compression should be used to compensate for its effects [48]. However, experimental studies of the effects of compression speed on preferences and speech intelligibility have given somewhat inconsistent results, and some have shown deleterious effects of fast compression [49–51]. As a result, many manufacturers use (primarily) slow compression.

Some deleterious effects of fast compression are as follows:

- For speech in background noise at a positive speech-to-noise ratio (which is the case for many everyday situations [52]), fast compression amplifies the background noise during brief dips in the speech [53], making the situation seem noisier than when slow compression is used.
- In reverberant situations, fast compression leads to amplification of the low-level “tails” of the reverberation, exacerbating deleterious effects of the reverberation [54, 55].
- Multiple sounds whose envelopes are uncorrelated at the input to the hearing aid (e.g., two talkers, or a talker with music in the background) have partially correlated envelopes after the application of fast compression, because the same-time-varying gain is applied to all sounds in a given channel. This effect is called cross-modulation, and it can lead to reduced speech intelligibility and poorer sound quality [51, 56, 57].

Some limitations of slow compression are as follows:

- Loudness recruitment makes amplitude-modulated sounds appear to have a greater modulation depth than “normal” [48] and slow compression does not compensate for this. As a result, dynamic fluctuations in level of the notes played by a musical instrument appear exaggerated: some notes may appear to “jump out” [58]. Similarly, some speech sounds may “jump out,” an effect that can be appreciated by people with normal hearing when listening to simulations of hearing loss [59, 60].
- A related effect is that for a plucked instrument like a guitar, the notes appear to fade away more rapidly than “normal”; what should sound like a gradually decaying note is heard more like a pizzicato note.
- When an intense sound suddenly ceases, or when there is an abrupt decrease in level, it takes the gain some time to recover, so soft sounds may be inaudible for a while. This is primarily the consequence of a long release time. I have experienced such effects with several hearing aids, for which the gain recovers over a period of several seconds. This effect can be reduced, but not eliminated, by AGC systems using multiple time constants [61, 62].

The problems associated with fast compression arise mainly because the same time-varying gains are applied to all sounds reaching the hearing aid. A potential solution is to separate the mixture of sounds into independent sources and to apply compression separately to each source [63, 64]. One approach to this problem has been described by Zhang et al. [65, 66]. They used a deep neural network (DNN) to segregate the speech of a target talker from background sounds (e.g., babble, traffic noise, cafeteria noise, sirens) and to apply fast compression to the target speech and slow compression to the background sounds. The compressed signals were combined with a controllable amount of background reduction, chosen to be 15 dB in preliminary experiments. Based on objective metrics, the Hearing Aid Speech Perception Index (HASPI) [67] and the Hearing Aid Speech Quality Index (HASQI) [68], the DNN-based methods gave better outcomes than comparison systems that used similar noise reduction but were based on slow or fast compression [65, 66] or on a signal-to-noise ratio (SNR)-aware system, which used fast compression when the SNR was high and slow compression when the SNR was low [69]. Evaluations of sound quality using hearing-impaired participants also showed superior results relative to comparison systems based on slow or fast compression or SNR-aware compression [65]. Such an approach could be implemented in hearing aids in the near future, since the time delay produced by the DNNs of Zhang et al. [65, 66] was only about 10 ms, and the computational requirements of the DNNs were comparable to those of the most powerful DNN already in use in hearing aids. However, extending the approach to situations with multiple talkers of a similar level remains challenging.

Overall, based on the undesirable side effects of both fast-acting and slow-acting compression and the limited effectiveness of current compression systems in compensating for reduced dynamic range, I give a rating for the compression systems currently used in hearing aids of 6/10.

## Feedback Cancellation

For moderate-to-severe high-frequency hearing losses, the gain prescribed by hearing aid fitting formulas [23–25] might lead to acoustic feedback (howling or whistling) at high frequencies. Such feedback occurs when the sound produced by the hearing aid leaks back to the hearing aid microphone and sets up a sustained oscillation. Feedback is a greater problem with an open fitting than with a closed fitting; the former might be used for a person with near-normal low-frequency hearing, in order to reduce the occlusion effect. Most modern hearing aids incorporate systems for reducing or cancelling acoustic feedback. These usually have two stages: (a) a “static” system based on in situ measurement of the feedback path (the transfer function from the hearing aid receiver to the hearing aid microphone) during fitting of the hearing aid and (b) an adaptive system to deal with changes in the feedback path, produced, for example, by a nearby wall or by holding a telephone near the ear [70]. These systems work by using the estimated feedback path to predict the unwanted feedback components reaching the microphone signal. A cancellation signal is then generated to eliminate the unwanted components via destructive interference. Sometimes a small frequency shift is introduced to decorrelate the input and output of the hearing aid, which aids estimation of the feedback path [71].

Feedback cancellation systems have been greatly improved in effectiveness since they were first introduced, and they allow greater gains to be achieved than would be possible without such cancellation. They also allow the use of more open fittings, which can be useful for alleviating the occlusion effect. However, they can still introduce artifacts of various types that degrade sound quality. The artifacts are usually short-lived and not very noticeable for speech, but may be more marked for music. The artifacts include the following:

- Transient whistling sounds when the acoustic conditions change.
- Steady tones may sound like amplitude-modulated tones.
- Steady tones may sometimes be cancelled.
- An “after tone” may be heard when a steady tone ceases.

In addition, peaks in the frequency response occur at frequencies where the gain is just below the gain that would lead to sustained feedback. This makes the sound appear colored and adversely affects sound quality [72]. Processing using DNNs can alleviate this problem because the DNNs can be trained to reduce the coloration as well as to cancel the feedback [26, 73].

Overall, I give a rating for the feedback cancellation systems currently used in hearing aids of 7/10.

## Programs and Automatic Program Selection

Many hearing aids allow the use of multiple programs that are intended to be optimal for use in specific listening situations, such as speech in quiet, speech in background noise, or music [74]. The hearing aid settings for each program are either set to the manufacturer’s default values or are set by the person fitting the hearing aid, using the manufacturer’s software. However, it is not always obvious how the hearing aid settings should be adjusted to suit different listening situations [75], and recommendations can differ across manufacturers. While it seems obvious that noise-reduction systems (discussed later in this paper) should be activated for listening to speech in noise, it is not obvious how to set the “strength” of the noise reduction. Also, it is not obvious how a “speech in quiet” program should be modified for listening to music. Common recommendations for music are to use a more extended low-frequency response, to use slow-acting compression or less compression, and to deactivate or reduce the strength of feedback cancellation [76].

Most modern hearing aids incorporate a program labeled “automatic,” which selects the hearing aid settings automatically according to the identified situation or “scene” [74]. In some hearing aids, a DNN is used to map from the acoustic features of a scene to the appropriate hearing aid settings, and this may be done using small step-wise changes in several parameters rather than simply selecting parameters appropriate for the most closely matching “scene.” The automatic program is usually set as the default program that is activated when the hearing aid is turned on, and it is likely that many users leave their hearing aids in the default automatic setting most if not all of the time. While there is evidence for small benefits of automatic program selection under simplified and artificial listening situations [77], there is a dearth of information about the accuracy and effectiveness of automatic scene classifiers in real-life situations. In my experience, they are far from perfect, although it must be acknowledged that scenes themselves are often ambiguous. For example, music together with multiple talkers might be “undesired” background music in a restaurant, in which case a directional microphone setting might be appropriate, or it might be desired music in a jazz club, in which case an omnidirectional setting might be appropriate.

Overall, I give a rating for the effectiveness of scene classification and automatic classifiers of 5/10 to 8/10, the range reflecting the dearth of published work evaluating these systems. A rating of 5/10 would be applicable to scene classifiers that were inaccurate or that did not set the hearing-aid parameters appropriately for the selected scene, while a rating of 8/10 would be appropriate for more accurate classifiers that generally selected hearing-aid parameters that were largely appropriate for the scene.

## Wireless Connectivity

Many hearing aids can communicate wirelessly with remote devices, including the following:

- Mobile (cell) phones: the phone signals are transmitted directly to the aid(s). This gives improved quality and clarity, especially with a closed fitting, and is a feature that is highly rated by users [19]. The improvements occur because: (1) the (usually not great) phone loudspeaker is bypassed; (2) background sounds are not present in the transmitted signal (except to the extent that such sounds are picked up at the “far” end); (3) the target speech is presented to both ears while any background sounds differ somewhat at the two ears, allowing some benefits of binaural listening [78]. Calls can be accepted and ended by a gesture such as double tapping of the ear. The microphones on the hearing aid(s) are often used to pick up the user’s voice, meaning that the phone can be left in a pocket or purse.
- A remote microphone: this can be placed near the target talker (via a necklace or clip) or on a table. Some remote microphone systems include multiple microphones that can be used to enhance the voice of the most prominent talker or to enhance a voice coming from a specific direction. This can be helpful for listening in restaurants or lectures, although the benefit is limited when an open fitting is used because of the leakage of background sounds through the vent. Also, such systems do not solve the problem of selecting the direction of the “target” talker (the talker that the user wants to listen to) when several people are talking at once.
- A television: direct streaming reduces the effect of room reflections and allows personalized adjustment of volume.
- Auracast: this is a recent feature of the Bluetooth standard that allows a device to broadcast high-quality audio to an unlimited number of nearby receivers, including hearing aids, without a pairing process. It can enable hearing-aid users to hear audio from TVs and public address systems in places like airports, railway stations, and lecture halls. For hearing aid users, Auracast may be used via a mobile (cell) phone, which can allow the user to select the broadcast that they want to hear from a list.

In addition, the phone can be used as a remote control to change programs, volume, and settings such as directionality.

Overall, I give a rating for the wireless connectivity systems currently used in hearing aids of 10/10 when a closed fitting is used and 7/10 when an open fitting is used. The lower rating for an open fitting reflects two factors: (1) sounds that are transmitted to the hearing aid will sound “tinny” because of the lack of low-frequency sound components in the output of the aid; and (2) the clarity of the transmitted sounds will be reduced by ambient sounds leaking through the vent.

## Stability of Output

Many hearing aid users (including me) complain that the output of their hearing aids is not stable; the output seems to vary from time to time, especially at high frequencies. There are several possible sources of instability:

- The eartip may not be properly positioned. This can occur with flexible (non-custom) domes as a result of improper insertion or jaw movements (the ear canal is flexible). It can also occur with custom earmolds as a result of jaw movements or a loose fit. A tighter fit might be more stable but could lead to discomfort. Even a small misalignment can transform a closed fitting to effectively an open fitting.
- For direct-drive systems like the Earlens, the positioning of the coil in the eartip (which transmits power and signal) may vary relative to the eardrum-mounted receiver coil, leading to variable signal transmission.
- Wax in the vent. This increases the occlusion effect, since the sound generated by the vibration of the walls of the ear canal in response to the user’s own voice cannot escape.
- Blocking of the wax guard that is intended to protect the receiver from wax. This is a common problem, and it reduces the output of the hearing aid, especially at high frequencies. While a hearing aid dispenser will usually instruct the client on when and how to change the wax guard, some users may forget to do this. Also, wax may accumulate gradually, which makes it harder to know when the wax guard should be changed.
- The microphone ports may become partially clogged with dirt and grease, an effect that tends to reduce the high-frequency output and builds up gradually.

It should be possible for hearing aid manufacturers to design systems for detecting automatically when the wax guard needs to be changed or when the microphone ports need to be cleaned and alerting the user via a warning signal delivered via the hearing aid receiver. This could be done, for example, by detecting when the user is talking (many hearing aids have own-voice detection systems), and assessing whether the spectral shape of the sound corresponds to “normal”; partial or complete blocking of the wax guard or the microphone ports would result in a greater reduction of high-frequency energy than of low-frequency energy. However, to my knowledge, no commercial hearing aid incorporates such a feature, although some hearing aid manufacturers have incorporated “troubleshooting” features in the hearing aids and/or in a phone app to help solve some of the problems discussed in this section.

Research on alleviating these effects is not exciting, but it is of practical importance, since problems of this type are common, especially among older people. Overall, I give a rating for the stability of the output of hearing aids of 5/10, on the basis that instability is an ongoing problem that has yet to be adequately addressed.

## Beamformers

A common complaint of hearing-impaired people is difficulty understanding speech in noise [79, 80]. Hearing aids can alleviate this problem using various forms of noise reduction. One approach uses the output from two or more microphones (four if signals are swapped between bilaterally fitted aids) to create a directional characteristic [74]. Such systems are often called “beamformers,” by analogy with a beam of light. This approach can be effective when the noise sources come from a different direction to that of the speech [81]. The “beam” can be pointed towards the most prominent sound source or (more commonly) towards the front. This helps if it is possible to look towards the source of interest. Otherwise, it makes the speech-to-background ratio worse, for example, at a restaurant when looking at a plate rather than at the desired talker, or at a poster session at a conference, when looking at a poster rather than the presenter. Even in small-group conversations, a listener sometimes looks away from a target talker [82]. Also, people move their eyes as well as their head when a new target talker starts: on average older people do 57% of the movement with their head and the rest with their eyes, so there is a mismatch between the hearing aid beam direction and the direction of the target talker [82].

Any benefit of beamformers is markedly reduced when an open fitting is used, since background sounds from all directions can leak through the vent, diluting the directional effect, although some benefit is still obtained [83, 84]. A possible solution is to use an eartip fitted with an “active vent” [85], which is electrically operated by the hearing aid; the vent is open in quiet situations, hence alleviating the occlusion effect, but closes when the listening situation is identified as “speech in noise” and the sound level is high, thereby preserving the benefit of the beamformer. It is assumed that the occlusion effect will be less noticeable in situations where there is a high level of background noise or that the benefits of the beamformer outweigh the deleterious occlusion effect. When using such a system, I found two limitations of the active vent. Firstly, it did not always select the open or closed setting appropriately for the situation (I could tell when it switched settings because it made a “cracking” sound each time it switched). Secondly, the occlusion effect was still bothersome, because when the background noise level was high, I raised the level of my voice accordingly, and my voice sounded unpleasantly boomy.

Beamformers are also used in remote microphone systems that transmit signals wirelessly to hearing aids [86]. This can increase the ratio of target speech to background noise at the output of the remote microphone, increasing the benefit that it provides.

Overall, I give a rating for beamformers in hearing aids of 6/10 for a closed fitting and 3/10 for an open fitting.

## Noise Reduction Based on DNNS

Single-microphone noise reduction (usually independent at the two aids when bilateral aids are used) has been employed in hearing aids for many years. Early systems were mainly based on analysis of the patterns of amplitude modulation in different frequency channels. Speech is characterized by strong low-frequency amplitude fluctuations. Gains were kept unaltered for channels with such fluctuations, which were assumed to be dominated by speech, while gains were reduced for channels in which the amplitude fluctuations were less strong or had a high rate [87]. These systems were of limited effectiveness [88, 89]*.* More recent systems have used DNNs (based on the output of one or more microphones) intended to improve the intelligibility of speech in background sounds [90–92]. DNNs provide greater improvements in both intelligibility and listening comfort than traditional methods, partly because DNNs are very effective in performing mapping operations (mapping from noisy speech to “clean” speech), provided that sufficient training data are available [92].

Ideally, a DNN should preserve the voice of the target talker while attenuating other sounds, including other talkers. However, it is very difficult to devise methods for determining who the target talker is and to do this accurately and with low latency. Several approaches have been tried [93–96] but none has yielded a practical solution that works well in a range of everyday situations.

Given that the “ideal” solution is not yet possible, several different approaches to training DNNs have been adopted, depending on the manufacturer. The DNN may be trained to:

- Preserve all speech sounds, regardless of direction, and to attenuate non-speech sounds.
- Preserve the voice of the most prominent talker, regardless of direction, and attenuate all other sounds, including other talkers.
- Enhance the voice of a talker coming from the front (combined with directional processing).

Approach (c) suffers from the same problem as for beamformers, namely that the ability to hear the target talker will be impaired whenever the user looks away from the target talker. Approach (a) is of limited benefit in “cocktail party” situations. I have been using a hearing aid with a DNN based on approach (b) for about 15 months (I only use one hearing aid, since I am completely deaf in one ear). I programmed the aid so that I could manually activate the DNN processing (via the mobile phone app) when desired and I set the directionality to a low value, so that the most prominent talker would be emphasized even when coming from the side or back.

My hearing loss is mild at low frequencies, so initially I used the DNN-based hearing aid with an open fitting. With this fitting, the benefit of the DNN processing was limited, because in high-noise situations too much background sound leaked through the vent. I then tried a more closed fitting (with a very small vent), which led to higher but bearable occlusion effects. With the more closed fitting, the benefit of the DNN processing was markedly increased, especially in situations with one dominant talker. The benefit seemed to be equivalent to an improvement in the signal-to-background ratio of about 4 dB. Examples of situations where a benefit occurred are as follows:

- In a restaurant at a table with four people, where usually only one person was talking at a time, but people at other tables were also talking.
- In a poster session at a conference when standing near the presenter, even when looking at the poster rather than the presenter.
- In a room with high reverberation (the DNN partially suppressed the echoes), presumably because some backgrounds including reverberation were used during the training of the DNN.

The benefit from the DNN processing was much less when two or more people with similar levels were talking at the same time.

Overall, I give a rating for DNN processing based on approach (b) of 8/10 for a closed fitting and 5/10 for an open fitting, on the basis that the DNN processing is effective in many (but not all) situations with a closed fitting, but the benefit is reduced with an open fitting (as is also the case for other methods of noise reduction).

## Avenues for Dealing with Problems Associated with Open-Fittings

Many hearing aid users have only mild hearing loss at low frequencies and prefer an open fitting to reduce occlusion (and avoid high humidity in the ear canal) [19]. One of the deleterious effects of open fittings is comb-filtering, which was described earlier. Peaks in the frequency response produced by comb-filtering occur at frequencies corresponding to the reciprocal of integer multiples of the delay. For example, if the delay is 5 ms, peaks in the frequency response occur at integer multiples of 200 Hz. If the delay is reduced to 0.5 ms, as is the case for one manufacturer of hearing aids, the peaks in the frequency response occur at 2000 Hz and above, a frequency where most hearing aids produce a gain greater than 10 dB and the ripples in the frequency response are small. Thus, making the delay very small effectively decreases the comb-filtering effect. However, the use of a very short delay limits the type of signal processing that can be done. For example, most DNNs require delays of several ms to work effectively.

An alternative approach is to use a closed fitting combined with active occlusion cancellation [85, 97]. This approach requires a microphone facing the ear canal, which detects the mixture of sounds generated by the hearing aid and by vibrations of the walls of the ear canal in response to the user’s own voice. The sound in the ear canal generated by the hearing aid is partly predictable from the signal picked up by the external microphones, and this allows the generation of a signal to cancel the sound generated by the user’s voice, via destructive interference, while preserving the desired signal. Such an approach not only reduces the occlusion effect but also preserves the benefits of directional processing and noise reduction. While this approach has given promising results in laboratory studies, it has not, to my knowledge, been incorporated in commercial hearing aids, although it is available in some earbuds that can act like hearing aids.

**Fig. 2:**
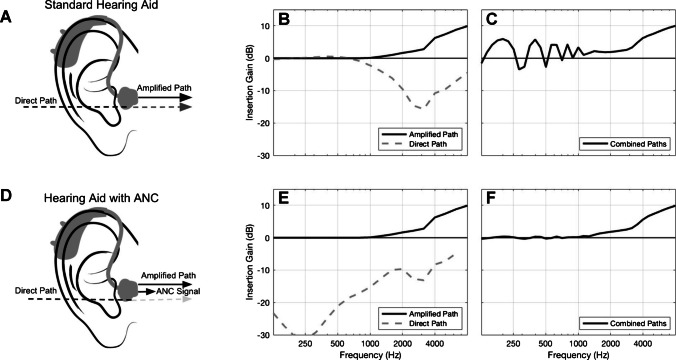
Illustration of the approach used for active noise cancellation and active cancellation of sound leaking through the vent of a hearing aid. From [98], with permission of the first author

A related but different approach to alleviating problems associated with open fittings is to keep the fitting open but to cancel the signal leaking through the vent [98]. This approach is similar to that used in many commercially available earphones and earbuds, where it is called active noise cancellation (ANC). The approach is illustrated in Fig. 2 for a hypothetical user with a mild high-frequency hearing loss. As for occlusion cancellation, a microphone facing the ear canal is used to pick up the sound in the ear canal.

The top part of Fig. 2 illustrates the operation of a standard open-fitting hearing aid. Panel B shows the gain for the amplified path (solid line) and for the sound leaking through the vent (direct path, dashed line). Low frequencies leak through the vent almost without attenuation, whereas high frequencies are attenuated. The resulting comb-filtering is shown in panel C. For illustrative purposes, it was assumed that the hearing aid has a gain of 0 dB for all frequencies below 1000 Hz. In practice, the gain would become negative at low frequencies, and the comb-filtering would be most prominent over the frequency range from about 500 to 1000 Hz. The bottom part of Fig. 2 illustrates the operation of a hearing aid with ANC. The gain for the amplified path (solid line in panel E) is the same as for the standard aid, but the gain for the direct path is markedly reduced at low frequencies, where ANC is most effective. The resulting gain in the ear canal (panel F) varies smoothly with frequency; the comb-filtering is eliminated. In principle, ANC can preserve the benefits of directional processing and noise-reduction systems, which would otherwise be reduced by the leakage of sound through the vent of an open fitting. ANC might affect perception of the user’s own voice, but this has not, to my knowledge, been systematically studied. Approaches using ANC have not, to my knowledge, been implemented in hearing aids.

## Conclusions

Hearing aids have improved markedly over the last 20 years, especially with regard to connectivity, directional processing, and feedback cancellation. However, there is still room for improvement in the frequency range over which the gain is sufficient to restore audibility, the operation of multi-channel compression systems, the comb-filtering associated with open fittings, the benefit of directional processing and noise reduction when open fittings are used, and the occlusion effect when closed fittings are used. For those with mild hearing loss at low frequencies, problems with occlusion may be solved by a closed fitting combined with active occlusion cancellation or an open fitting combined with direct sound cancellation. DNN processing has great potential for enhancing speech in noise, but the (tough) problem of selecting the target talker remains to be solved.

## Data Availability

Not applicable.
